# A Century of Medical History in the County of Erie.—1800–1900

**Published:** 1898-09

**Authors:** William Warren Potter

**Affiliations:** Buffalo, N. Y.


### A CENTURY OF MEDICAL HISTORY IN THE COUNTY OF ERIE.—1800-1900.

By WILLIAM WARREN POTTER, M. D., Buffalo, N. Y.

*Pioneer Physicians—Medical Societies—Medical Colleges—Hospitals—Medical Journals—Medical Officers of the Civil War—Women Physicians—History of Homeopathy—Individual Members of the Profession.*

[*Continued from the August edition.*]

1886--J. W. Grosvenor, H. W. Bode, F. M. Rich, D. A. Morrison, DeLancey Rochester, J. M. Stanley, J. G. Whitwell, E. E. Johnson, Dewitt C. Greene, William C. Callanan, Thomas M. Crowe, Arthur W. Hurd, E. H. Norton, E. T. Smith, Mark M. Brooks,

William L. McFarland, H. H. Bingham, Benjamin W. Cornwell, T. F. Dwyer, Elmer Starr, Edward L. Gager, John T. Pitkin.

Officers for 1886—President, E. T. Dorland; vice-president, O. C. Strong; secretary, William H. Thornton; treasurer, F. W. Abbott; librarian, J. B. Samo; censors, Edward Storck, Henry R. Hopkins, A. H. Briggs, P. W. Van Peyma, William H. Gail; delegates to state medical society, B. Bartow, F. S. Crego, M. B. Folwell, F. W. Hinkel, C. W. Bourne.

1887—George H. Westinghouse, E. J. Murphy, W. E. Jennings, Gustave Pohl, C. J. Hill, W. E. Robbins, E. M. Wetherill, E. T. Stevens, C. G. Steele, Harry A. Wood, Jacob Goldberg, Bina A. Potter, G. W. Cutter, William A. Hoddick, Thomas G. Allen, George S. Palmer, J. G. Meidenbauer, R. E. Miller, Julius Pohlman.

Officers for 1887—President, O. C. Strong; vice-president, J. D. Hill; secretary, William H. Thornton; treasurer, F. W. Abbott; censors, Edward Storck, Henry R. Hopkins, William H. Gail, P. W. Van Peyma, Charles H. Wetzel.

1888—B. M. Strong, W. H. Bergtold, William H. Heath, J. J. Birmingham, Charles W. Howell, Charles E. Congdon, Ernest Wende, M. B. Cook, M. B. Searls, H. G. Matzinger, Paul F. Bussman, W. Scott Renner, W. M. Ward, Bernard Cohen, Jacob M. Falk, John Ketchum, S. Goldberg, B. F. Rogers, W. T. Tanner.

Ernest Wende, who became a member in 1888, was appointed health commissioner of Buffalo under the new charter, January 1, 1892. Under his administration many reforms have been instituted, and the death-rate of Buffalo has become the lowest of any city of its size in the world. He was reappointed for a term of five years by Mayor Edgar B. Jewett, to take effect January 1, 1897.

Officers for 1888—President, John D. Hill; vice-president, Rollin L. Banta; secretary, William H. Thornton; treasurer, F. W. Abbott; librarian, J. B. Samo; censors, Edward Storck, Henry R. Hopkins, P. W. Van Peyma, A. R. Davidson, W. D. Greene.

1889—Westervelt Banta, J. N. Goltra, George F. Cott, Electa B. Whipple, John D. Flagg, H. C. Buswell, Clark F. Bruso, Ira C. Brown, Fridolin Thoma.

Officers for 1889—President, R. L. Banta; vice-president, G. W. McPherson; secretary, W. H. Thornton; treasurer, F. W. Abbott; librarian, James B. Samo; censors, Edward Storck, P. W. Van Peyma, Thomas Lothrop, Henry Lapp, Henry R. Hopkins; delegates to the state medical society, A. E. Persons, Roswell Park, E. L. Bissell, F. W. Bartlett, E. H. Long.

1890—L. L. Ball, A. L. Benedict, J. D. Bowman, John J. Champlin, M. A. Crockett, Sydney A. Dunham, C. E. Ernest, Howard L. Hunt, J. M. Krauss, C. B. LeVan, George W. T. Lewis, George

H. McMichael, John Middleton, R. S. Myers, E. N. Pfohl, T. Haven Ross, Clinton A. Sage, George H. Sisson, C. M. Smith, T. S. Stewart, John J. Twohey, W. Wolff, F. B. Voght, J. E. Whitmore, E. E. Briggs, F. M. Gipple, Allen A. Jones, William C. Krauss, R. E. Moss, M. Retel, Emil Schroeder, Hugo Schmidt, Otto Thoma, J. C. Thompson.

Officers for 1890—President, G. W. McPherson; vice-president, E. C. W. O'Brien; secretary, William H. Thornton; treasurer, F. W. Abbott; librarian, J. B. Samo; censors, Edward Storck, Thomas Lothrop, Henry Lapp, Joseph Haberstro, P. W. Van Peyma.

1891—L. B. Dorr, B. S. Bourne, Henry J. Mulford, E. A. Forsyth, Henry Y. Grant, C. T. Wolsey, E. A. Milring, William A. P. Andrews, R. L. Patteson, Walter J. Riehl, E. G. Danser, William Dowlman, H. S. Townsend, William P. Clothier, John J. McCullough, John Hausberger, E. H. Young, William H. Chace, C. R. Jennings, J. F. Sell, M. J. O'Connell, J. P. Wilson, William H. Woodbury.

Officers for 1891—President, E. C. W. O'Brien: vice-president, William H. Gail; secretary, William H. Thornton; treasurer, F. W. Abbott; librarian, Lucien Howe; censors, Edward Storck, R. L. Banta, Henry Lapp, M. B. Folwell, E. H. Ballou; delegates to the state medical society, B. H. Grove, B. H. Daggett, Edward Clark.

1892—W. H. Baker, C. A. Schladermundt, Harriet E. Sheldon, F. H. Powell, Charles H. Meahl, E. H. Tweedy, Charles H. Woodward, Charles P. Clark, Lillian C. Randall, A. B. Knisley, Lewis C. Smith, Irving W. Potter, Arthur B. Allen, Charles J. Reynolds, John R. Gray, F. L. Watkins, Frederick Preiss, F. E. Hill, W. J. Beutler, J. M. Hewitt, A. J. Colton, M. V. Ball, J. J. Drake, L. A. Denton, Mary I. Denton, H. U. Williams, J. T. Harris, E. J. Gilray, C. B. Johnson, A. N. Collins, A. G. Bennett.

Officers for 1892—President, William Warren Potter; vice-president, William H. Gail; secretary, E. H. Long; assistant-secretary, Ira C. Brown; treasurer, Edward Clark; librarian, Charles A. Ring; censors, Edward Storck, Henry R. Hopkins, P. W. Van Peyma, J. J. Walsh, Henry Lapp; delegates to state medical society, B. H. Grove, Edward Clark, E. C. W. O'Brien, Ernest Wende, H. E. Hayd.

At the semi-annual meeting, June 14, 1892, Drs. Joseph Price, of Philadelphia, Charles A. L. Reed, of Cincinnati, Lewis S. McMurtry, of Louisville, James F. W. Ross, of Toronto, and Brooks H. Wells, of New York, attended and read papers, or took part in the discussions. This meeting was probably the most remarkable in the history of the society. Never before had so many distinguished men attended from a distance to participate in the proceedings. The papers and the discussions were published in full in the BUFFALO MEDICAL JOURNAL for August and September, 1892, and reprinted in separate form.

1893—Eleanor McAllister, Loren H. Staples, Carlos E. Bowman, Mary T. Greene, H. C. Leonhardt, Jane W. Carroll, Robert S. Hambleton, Frank J. Thornbury, Henry T. Carter, Alfred E. Diehl, Alfred F. Erb, Edward L. Frost, Franklin C. Gram, George J. Hearne, George A. Himmelsbach, H. Corwin Jones, Charles E. Long, Edward J. Myer, Ferdinand G. Moehlau, Duncan Sinclair, James Stoddart, Clarence A. Tyler, G. W. Wende, J. F. Whitwell, Edward R. Wiser.

Officers for 1893—President, John Parmenter; vice-president, William H. Jackson; secretary, E. H. Long; assistant-secretary, George F. Cott; treasurer, Edward Clark; assistant-treasurer, E. A. Smith; librarian, Charles A. Ring; censors, P. W. Van Peyma, Henry Lapp, J. F. Krug, J. H. Potter, A. L. Benedict; delegates to the state medical society, William H. Bergtold, J. J. Walsh, William C. Callanan.

1894—Albert T. Lytle, Ada C. Lathrop, Harry Mead, Dewitt H. Sherman, Horace Clark, Charles S. Jewitt, A. W. Bayliss, C. S. Siegfried, Francis T. Metcalfe, Ludwig Schroeter, William G. Taylor, Helen J. C. Kuhlman, William Meisberger, Maud J. Frye, William C. Fritz.

Officers for 1894—President, William H. Gail; vice-president, F. W. Bartlett; secretary, F. C. Gram; assistant-secretary, George F. Cott; treasurer, Edward Clark; assistant-treasurer, Eugene A. Smith; librarian, William C. Callanan; censors, J. F. Krug, James S. Porter, Henry Lapp, A. L. Benedict; delegate to state medical society to fill vacancy, J. H. Pryor.

1895.—N. Victoria Chappell, A. H. Macbeth, G. P. Hepp, Charles A. Clemens, William G. Bissell, A. T. O'Hara, Clara E. Bowen, Evangeline Carroll, Walter M. Kidder, F. H. Field, Thomas B. Carpenter, Homer J. Grant, Hiram A. Kendall, Lawrence J. Hanley, P. H. Hourigan.

Officers for 1895—President, F. W. Bartlett; vice-president, J. G. Thompson; secretary, Franklin C. Gram; treasurer, Edward Clark; censors, J. B. Coakley, M. Hartwig, E. H. Long, F. T. Metcalfe, Henry Lapp.

1896.—R. H. Lounsbury, John V. Woodruff, F. H. Milliner, Amelia F. Dresser, Jacob Miller, C. E. Fisher, Ray H. Johnson, John E. Bacon, H. C. Roth, Martha F. Caul, Wellington G. Grove, Richard H. Satterlee, J. Grafton Jones, John B. McArtey, C. T. Stewart, E. E. Blaauw, Henry Osthues and J. Henry Dowd.

Officers for 1896—President, J. G. Thompson; vice-president, Henry R. Hopkins; secretary, F. C. Gram; treasurer, Edward Clark; censors, J. B. Coakley, M. Hartwig, B. G. Long, F. T. Metcalfe, Henry Lapp.

At the annual meeting, held January 14, 1896, the society having completed its 75th year, celebrated its diamond jubilee. Papers commemorative of the occasion were read by Drs. John Hauenstein, C. C. Wyckoff, John Cronyn and Franklin C. Gram. These papers were published in the BUFFALO MEDICAL JOURNAL during the next few months, and also were reprinted and sent out in pamphlet form to libraries and medical societies throughout the state.

1897—Jane North Frear, Frederick W. Hayes, Edward E. Koehler, Earl P. Lothrop, E. T. Rulison, A. E. Woehnert, Marion Marsh, Cora Billings Lattin, Henry W. Lattin and J. Glen Ernest.

Officers for 1897—President, Henry R. Hopkins; vice-president, Hiram P. Trull; secretary, Franklin C. Gram; treasurer, Edward Clark; librarian, William C. Callanan; censors, John B. Coakley, Charles E. Congdon, Thomas F. Dwyer, Irving W. Potter and Gustave A. Pohl.

At the semi-annual meeting, held June 8, 1897, Dr. Hopkins and Dr. Trull having declined to serve, Dr. Lucien Howe was elected president and Dr. John B. Coakley was elected vice-president for the remainder of the year.

1898—Renwick R. Ross, Henry M. Reinhardt, Julius Ullman, Lorenzo Burrows, Katherine S. Munhall, Mary M. Huntley, Edward M. Dooley, William Preiss, D. J. Constantine, L. E. McC. Pomeroy, Francis E. Fronczak, Carro Julia Cummings, John J. Finerty, F. W. McGuire and John H. Daniels.

Officers for 1898—President, Lucien Howe; vice-president, John B. Coakley; secretary, Franklin C. Gram; treasurer, Edward Clark; librarian, William C. Callanan; censors, John B Coakley, Charles E. Congdon, Thomas F. Dwyer, Irving W. Potter and Julius F. Krug; delegates to the state society, Herbert U. Williams, Arthur W. Hurd, Peter W. Van Peyma, Thomas B. Carpenter, Grover W. Wende, Eugene A. Smith, Earl P. Lothrop and J. G. Thompson.

Every possible effort has been made to verify the facts and dates given in the foregoing section, sometimes at considerable expenditure of time and patience, but they are believed to be correct in the main and are offered as containing much of interest to physicians as well as to many outside the ranks of the profession.

The society is now in flourishing condition, has 350 members and is contemplating the establishment of a medical home for itself and the other medical organisations in the county.

### BUFFALO MEDICAL ASSOCIATION.

The first attempt to organise a medical society with membership limited to the boundaries of Buffalo is recorded as having taken place July 16, 1831. On that date a constitution and by-laws for the medical society of the village of Buffalo was submitted for adoption. It comprised a preamble of three paragraphs, a constitution of twelve articles and a group of thirty-six by-laws. They were such as are usually adopted by medical societies and were signed by the following-named foundation members: Cyrenius Chapin, Judah Bliss, John E. Marshall, Josiah Trowbridge, Moses Bristol, Bryant Burwell, Henry R. Stagg, Alden S. Sprague, James N. Smith, Lucian W. Caryl, Orson S. St. John.

These physicians met again July 19, 1831, adopted the constitution and by-laws and elected the following-named officers: President, Cyrenius Chapin; vice-president, Judah Bliss; recording secretary, Bryant Burwell; corresponding secretary, Josiah Trowbridge; treasurer, Moses Bristol. Whereupon the organisation of the society was completed. Six members were chosen to read dissertations on specified subjects at successive meetings of the association. In accordance with this order at the next meeting, August 2, 1831, Dr. Caryl read a paper on the circulation of the blood, which was discussed by Dr. Trowbridge, in the course of which he referred to the case of Mrs. Gen. Porter, who had lately died. During 1831 five meetings were held, two papers were read, and three members paid fines of $2 each in default of presenting papers at designated times. The annual meeting was held January 2, 1832, when the following officers were elected: President, John E. Marshall; vice-president, Bryant Burwell; corresponding secretary, Josiah Trowbridge; recording secretary, Lucian W. Caryl; treasurer, Alden S. Sprague. Meetings were held February 7th and March 6th, but on April 3d, May 1st and June 5th, the secretary reported no quorum and recorded fines against delinquent members. This was the last attempt to convene the society and it died in less than a year after its organisation.

The second effort to organise within the lines previously mentioned was on January 22, 1836, when a meeting of the physicians and surgeons of the city of Buffalo was held at the office of Drs. Marshall and Harris. At this meeting Drs. Bryant Burwell, Marshall, Barnes, Hawley and Winne were appointed a committee to draw up a fee bill, and Drs. Miller, Salsbury, Sprague, McVickar and White were appointed a committee to prepare a constitution and by-laws for the proposed medical association. Another meeting was held January 27, 1836, at the same place, when Dr. Gorham F. Pratt was appointed chairman, and Dr. Charles Winne served as secretary. The committee on fee bill reported the following-named physicians as having pledged themselves to adhere to it: Bryant Burwell, Henry R. Stagg, James E. Hawley, John E. Marshall, C. H. Reynolds, Brock McVickar, Charles Winne, James P. White, Abraham Miller, Judah Bliss, Josiah Barnes, Alden S. Sprague and Francis L. Harris. The committee appointed to prepare the constitution and by-laws failed to report, hence no organisation was effected. It is stated as a reason for this failure that at the time mentioned everybody was carried away with the spirit of speculation engendered by the sudden prosperity of the times; hence, it was impossible to interest a sufficient number to maintain a medical organisation.

The third attempt, more successful because permanent, did not occur until ten years afterward. The BUFFALO MEDICAL JOURNAL, in its issue for July, 1845, which had lately been established printed the following notice: "*To the physicians of Buffalo:* Physicians of this city, members of the Erie County Medical Society, who are disposed to unite in forming a city medical society, are requested to meet at the office of Dr. Josiah Trowbridge, on Wednesday evening, July 2d, at 7 o'clock." The meeting was held has appointed, at which the following-named physicians were present: Josiah Trowbridge, Moses Bristol, Alden S. Sprague, George N. Burwell, John S. Trowbridge, Charles Winne, Josiah Barnes, Francis L. Harris, Horatio N. Loomis, H. M. Congar, Frank Hastings Hamilton and Austin Flint. Dr. Josiah Trowbridge was called to the chair and Dr. Flint was appointed secretary. A committee consisting of Drs. Loomis, Winne and Flint was appointed to prepare a constitution and by-laws. This committee reported at an adjourned meeting, held July 16, 1845, at the office of Drs. Sprague and Loomis. Its report was adopted after a debate during which some minor alterations were made. Officers for the ensuing year were elected as follows: President, Josiah Trowbridge; vice-president, Alden S. Sprague; recording secretary, Austin Flint. The constitution and by-laws were signed by Josiah Trowbridge, Moses Bristol, James P. White, Alden S. Sprague, H. H. Bissell, John S. Trowbridge, Sylvester F. Mixer, George N. Burwell, James B. Samo, Samuel G. Bailey, Austin Flint, Gorham F. Pratt, Francis L. Harris, H. M. Congar, William Treat, Silas Hubbard, Charles H. Wilcox and Josiah Barnes; total, eighteen members.

The first regular meeting of the Buffalo Medical Association was held at the office of Dr. Frank Hastings Hamilton, August 5, 1845, at 8 o'clock P. M. On this occasion Dr. Flint presented for inspection a heart with valvular lesions; Dr. Hamilton moved the appointment of a committee to collect statistics concerning shortening in fractured limbs. At the September meeting Dr. White presented a placenta with ossific deposit and reported a case of rupture of an ovarian cyst, caused by a fall, in which absorption of the fluid and recovery took place. We have mentioned the reports of these three men at these meetings because they, perhaps more than any others, gave direction to the early efforts of the society. Hereafter in these pages only such proceedings or acts of the association as may possess some general or public interest will be recorded. No reference to its scientific work will be made other than such as relates to public health, except when some question of paramount importance is presented.

At the meeting held December 3d, Drs. White, Barnes and Flint, as a committee, reported a fee bill, which was adopted and ordered printed for the use of members. At the meeting held March 3, 1846, the secretary applied for and obtained permission to publish the proceedings of the association in the BUFFALO MEDICAL JOURNAL. At this meeting Dr. Bryant Burwell introduced resolutions favoring a national medical convention, to be held at New York, May 1, 1846, and at a subsequent meeting Drs. Bryant Burwell and Alden S. Sprague, were elected delegates to attend the convention. At a meeting, held April 27, 1847, on motion of Dr. White, it was voted to raise the sum of $25 by subscription to help defray the expenses of the delegates to the American Medical Association. Dr. Josiah Trowbridge was elected delegate, but declined to serve; whereupon a committee was appointed to select a delegate, but subsequently reported that they had failed to do so.

At the annual meeting held August 3, 1847, the following-named officers were elected: President, Bryant Burwell; vice-president, C. H. Austin; secretary, William Treat. At a meeting held January 4, 1848, Dr. Sprague reported the successful amputation of a thigh while the patient was under the influence of ether and unconscious throughout the operation. This was the first capital operation performed in Buffalo under anesthesia. February 1st, Dr. Hamilton related the effects of chloroform upon himself. He obtained a specimen from Boston, of which he had inhaled about one ounce. The propriety of employing ether in obstetric practice was also discussed and an adverse opinion was elicited, though Dr. Loomis thought well of it in surgical operations if employed with proper care. April 4th, Dr. Hamilton offered a preamble and resolution providing for the painting of a portrait of Dr. Josiah Trowbridge, the first president of the association. The picture was subsequently painted by Wilgus, who received $68 for his work, and it was hung in the common council chamber November 7, 1848. Dr. George N. Burwell, at this meeting, reported seven cases of obstetrics in which he had used chloroform with entire safety and great comfort to the patients. Dr. Walter Cary having been chosen delegate to the American Medical Association was instructed to invite it to hold its next meeting in Buffalo.

On May 2, 1848, the by-laws were amended, providing that the annual election be held thereafter on the first Tuesday of April in each year. The object of this change was to afford an opportunity for the printing of the names of the officers in the city directory, which was then issued in June every year.

At the next annual meeting, August, 1848, the following-named officers were elected to serve until the ensuing April: President, Frank Hastings Hamilton; vice-president, S. F. Mixer; secretary, William Tréat. Primary board: Austin Flint, Walter Cary and H. M. Congar. The duty of the primary board was to examine students who desired to enter upon the study of medicine, and no physician in the capacity of preceptor was permitted to receive a student who did not possess the certificate of the primary board.

Annual meeting, April 3, 1849. The following-named were elected officers: President, Sylvester F. Mixer; vice-president, George N. Burwell; secretary, James M. Newman. May 1st, Dr. James B. Samo, from a committee appointed for that purpose, recommended the following as a minimum annual salary for medical services at the places named: Almshouse, $1,000; jail, $100; workhouse, $200. Fee in coroner's cases, $3 to $5; attendance on cholera hospital, $5 a day June 5th, Dr. Walter Cary reported a case of Asiatic cholera, the first in Buffalo during the epidemic then prevailing in this country.

June 10th. Special meeting. Dr. Josiah Trowbridge stated that the meeting had been called for the purpose of establishing a unformity in reporting cases of the prevailing epidemic cholera to the board of health; also to ascertain, by conference with the board of health, what in its opinion were cases of cholera and what it expected physicians to report as cases of that disease. Dr. C. C. Haddock and

Mr. A. McArthur, members of the board of health, were present. On motion of Dr. Flint it was ordered that a committee of three be appointed to draft a report expressive of the sense of the association relative to the object of the meeting. Drs. Flint, Treat and John S. Trowbridge were appointed as such committee to report at an adjourned meeting. The next morning after this meeting Dr. C. C. Haddock, in apparent good health, was attacked with diarrhea, which he neglected to heed, but pursued his public duties as health officer until late in the evening of the same day, at which time he was striken with cholera and died the next evening. July 12th the adjourned meeting was held in the common council chamber, when Dr. Flint read his report (published in the BUFFALO MEDICAL JOURNAL, August, 1849), and the death of Dr. Haddock was announced. Appropriate resolutions were passed in his memory.

April 2, 1850. The election resulted in the choice of the following-named officers: President, George N. Burwell; vice-president, Charles W. Harvey; secretary, William Ring. September 3d, Dr. Frank H. Hamilton read a biographical sketch of Dr. John E. Marshall. October 1st, Drs. Burwell, Pratt and Treat, were appointed a committee to prepare a biography of Dr. Cyrenius Chapin.

April 1, 1851, the following-named officers were chosen: President, Charles W. Harvey; vice-president, Silas Hubbard; secretary, James S. Hawley. Primary board: Drs. Wallis, Wyckoff and Treat. May 5th, Drs. Ring, Sprague and Hamilton were appointed a committee to obtain signatures to a petition to the legislature in favor of legalising dissection. Drs. Strong, Burwell and Congar were appointed a committee to memorialise the common council in relation to more perfect registration of deaths. At this meeting the association resolved to meet every fortnight instead of every month as heretofore.

April 6, 1852. Election of officers: President, C. W. Harvey; vice-president, Silas Hubbard; secretary, Sandford Eastman. Primary board: Drs. Wallis, Strong and Garvin. July 6, 1852, this being the evening for the obsequies of Henry Clay, on motion of Dr. Eastman the association adjourned for one week. August 3d. At this meeting sixty-two cases of cholera and thirty deaths were reported within the preceding week. Drs. Hamilton, Wilcox and Strong, were appointed a committee to report on the relation of upturning of soil to the causation of cholera.

April 5, 1853. Election of officers: President, Charles H. Wilcox; vice-president, James M. Newman. Primary board: Drs.

Eastman, Hawley and Ring. July 7th, Dr. Marshall Hall, of London, then visiting this country, attended the meeting of the association by invitation and proceeded to demonstrate the reflex nervous system of frogs. Afterward Dr. Flint entertained Dr. Hall and the members of the association at his home.

March 7, 1854. Dr. Sanford B. Hunt called the attention of the association to the propriety of taking observations of the temperature and humidity of the atmosphere, accentuating their importance in connection with disease. Means were at once taken to obtain proper instruments, which was the beginning of the valuable hygrometric observations that subsequently made Dr. Hunt so famous in this field of scientific inquiry.

April 4, 1854. Election of officers: President, James M. Newman; vice-president, P. H. Strong; secretary, Sanford B. Hunt. June 27th, Dr. Rochester reported a case of cholera that occurred June 12th, the first reported during this year. Dr. Hunt remarked on the epidemic of varicella at Hornellsville. There had been three hundred cases and ten deaths. August 1st, reports were made of cholera in Buffalo and Niagara Falls; it had been especially fatal at the latter place and Drs. Hunt and Fred Gardiner had each spent a week there, while Drs. Hamilton and Rochester had been several hours at Suspension Bridge. In the discussion Dr. Hunt remarked that the higher the dew point the more severe the disease.

February 6, 1855. Dr. Samuel G. Bailey gave a supper and social entertainment to the members of the association.

April 3, 1855. Election of officers: President, P. H. Strong; vice-president, Sandford Eastman; secretary, Sanford B. Hunt. Primary board: Drs. Hawley, Root and Baker.

BUFFALO MEDICAL AND SURGICAL ASSOCIATION.

At the meeting held in February, 1856, Dr. Baker, from the committee on organisation, reported a form for an act of incorporation. This was ratified April 1, 1856, when the Buffalo Medical Association became incorporated as the Buffalo Medical and Surgical Association.

April 1, 1856. Election of officers: President, Sandford Eastman; vice-president, Austin Flint; secretary, Sanford B. Hunt; treasurer, James M. Newman; librarian, William Howell.

April 7, 1857. Election of officers: President, Austin Flint; vice-president, C. C. Wyckoff; secretary, Sanford B. Hunt; treasurer, James M. Newman; librarian, B. H. Lemon. Primary board: C. L. Dayton, John Boardman, A. W. Nichols.

February 16, 1858, Dr. Rochester read an account of the illness of Dr. Morgan G. Lewis, who died of pyemia.

April 6, 1858. Election of officers: President, C. C. Wyckoff, vice-president, James M. Newman; secretary, Austin Flint, Jr.; treasurer, S. F. Mixer; librarian, B. H. Lemon. Primary board: Drs. Hawley, Eastman and King. Dr. White reported the successful restoration of the uterus after it had been inverted for six months. This operation had never before been successfully performed in this county. September 7th, Dr. White reported a case of inversion of the uterus of over fifteen years' standing, which he had restored to its normal place in fifty minutes, the patient being under the influence of chloroform. The woman died on the sixteenth day from general peritonitis. The autopsy, however, disclosed that the disease was a coincidence and not a sequence of the uterine restoration. There was no traumatism found as a result of the operation.

April 12, 1859.—Annual election. President, James M. Newman; vice-president, Thomas F. Rochester; secretary, Austin Flint, Jr., ; treasurer, C. B. Hutchins; librarian, B. H. Lemon.

August 2, Dr. Frank Hastings Hamilton expressed the opinion that a person during sleep could not be anesthetised by chloroform. This declaration gave rise to some discussion, most of the members coinciding with the views of Dr. Hamilton. December 6th, Dr. Hamilton introduced Dr. William K. Scott, an old and honored physician of Buffalo, for some years retired from the practice of his profession, yet who in his 72d year possessed so firm a nerve, an eye so undimmed, that he was daily in the habit of executing caligraphy in a manner most wonderful and pleasing. Dr. Scott stated that he had practised this exercise for the purpose of preserving and improving his eyes. He then presented for inspection 1,391 words written upon a circular card, 57-100 of an inch in diameter; each letter was beautifully and distinctly formed. Dr. Scott was president of the Medical Society of the County of Erie in 1844.

April 3, 1860. Election of officers: President, Thomas F. Rochester; vice-president, C. C. F. Gay; secretary, William Treat; treasurer, J. F. Miner; librarian, William Ring. Primary board: Frank H. Hamilton, Sandford Eastman, Henry Nichell.

April 2, 1861. Election of officers: President, C. C. F. Gay; vice-president, James P. White; secretary, J. F. Miner. August 6th, Dr. William Treat, who had just returned from Washington, where he had assisted in dressing the wounded that were brought into Fort Runyon, July 22d, after the battle of Bull Run, gave an account of that service. He stated that very few capital operations were made, as most of the wounded were able to walk. One amputation near the shoulder well dressed arrived from the field, and several soldiers with their forearms in temporary splints also came in on foot. The whole number dressed was about 125. Dr. Charles H. Wilcox, surgeon of the 21st regiment, and Dr. Joseph A. Peters, assistant surgeon, were actively employed, while Dr. Frank H. Hamilton, surgeon of the 31st N. Y., spent a portion of a forenoon at Fort Runyon. Dr. Treat went next day to the city hospital where he performed a similar service. September 3d, Drs. Gould, Samo and Ring, committee, presented resolutions upon the death of Dr. William Treat. This skilful physician, who had been a resident of Buffalo for twenty years, died soon after the last meeting of the association at which he gave an account of his service with the wounded at Fort Runyon.

April 1, 1862—Election of officers. President, James P. White; vice-president, H. M. Congar; secretary, J. F. Miner; treasurer, James B. Samo; librarian, William Gould.

At a meeting held January 6, 1863, Dr. P. H. Strong gave an account of his observations while on duty with the army in the field. He spent most of his time at Frederick, Md., in taking care of the sick and wounded after the battles of South Mountain and Antietam. He said the operations on the field were successfully, many times beautifully, done and that the wounded for the most part did well afterward. This statement applied especially to primary operations. His remarks were reported in full and received the commendation of the members present. At the annual meeting, April 7, 1863, the following-named officers were elected: President, H. M. Congar; vice-president, James B. Samo; secretary, J. F. Miner; treasurer, C. C. Wyckoff; librarian, J. B. Samo; primary board, Sandford Eastman, Henry Nichell, M. Shaw. The committee on fee bill, T. T. Lockwood and James P. White, reported in favor of increasing the fee for visits from one dollar to one dollar and fifty cents, and for office prescriptions from fifty cents to one dollar. This advance the committee affirmed was justified on account of the depreciation of currency and a proportionate rise in the price of the necessaries of life, whereupon the report was adopted.

At a meeting held May 5, 1863, Dr. White presented a specimen of extrauterine pregnancy, sent to him by Dr. Lauderdale, of Geneseo.

In commenting upon the condition Dr. White advocated abdominal section for the purpose of stopping hemorrhage and removing débris from the abdomen, and even before rupture he regarded the operation justifiable and he remarked that some way should be devised for saving women after this accident. Thus early did Dr. White foreshadow the present practice in such cases.

At a meeting of the physicians of Buffalo, held February 12, 1864, resolutions of respect to the memory of Dr. William H. Butler were adopted. Dr. Butler had been on duty as acting assistant surgeon at Armory Square Hospital, Washington, and his colleagues in that service sent a testimonial that was published in the BUFFALO MEDICAL JOURNAL with the proceedings of the meeting.

Annual meeting, April, 1864. Dr. James B. Samo was elected president; William Ring, vice-president; Joseph A. Peters, secretary; T. T. Lockwood, treasurer; T. M. Johnson, librarian.

Annual meeting, April, 1865. Election of officers: President, William Ring; vice-president, William Gould; secretary, Joseph A. Peters; treasurer, C. C. Wyckoff; librarian, James B. Samo.

August 1st. Dr. John S. Trowbridge presented a copy of the tables of measurements of men examined at the local Provost Marshal's office, which he as surgeon had been required to make and file in the Provost Marshal General's office at Washington. December 5th, Dr. Joseph A. Peters resigned the office of secretary and Dr. Thomas M. Johnson was elected to fill the vacancy.

February 6, 1866. Dr. Thomas F. Rochester introduced a resolution that was adopted, urging every member of the profession to attend a meeting February 13th, to take action respecting sanitary measures proper to be instituted for the welfare and protection of the citizens of Buffalo during the ensuing summer. The meeting was held on the date named, a committee appointed to confer with the mayor and board of health on the subject indicated, and an address of instructions sent out to the public.

Annual meeting, April 3, 1866. President, William Gould; vice-president, John S. Trowbridge; secretary, T. M. Johnson; treasurer, T. T. Lockwood; librarian, James B. Samo. July 3d, there were no communications to be read at the meeting, but the propriety of a revision of the constitution and by-laws was discussed. September 4th, the question was brought up in reference to druggists receiving a percentage on physicians' prescriptions, whereupon Dr. Miner expressed the belief that in Buffalo druggists were conducting an honorable business, and that he had not known either a respectable druggist to offer, or a physician to accept, such a division of profits. October 2d, Dr. Joshua R. Lothrop presented a paper in which he related a case of an alleged mistake of an apothecary at Lockport in putting up a prescription. Dr. Clark, of Lockport, the prescribing physician, had ordered fluid extract of uva ursa in doses of about twelve drops, but the apothecary put up fluid extract of veratrum viride. After taking two doses the patient was seized with alarming symptoms. Subsequently the patient sued the apothecary and obtained a verdict of $800 damages. The paper of Dr. Lothrop appears in full in the BUFFALO MEDICAL JOURNAL, October, 1866. December 4th,—a discussion was had over the propriety of appointing a member at each meeting to designate a subject for consideration at the next meeting and who should prepare himself to lead in such discussion. Dr. Miner was elected and initiated the course at the next regular meeting.

February 5, 1867. Dr. Miner opened a discussion on surgical remedies—vesicants, rubefacients, setons, issues and the like. Some of the members, among whom were Drs. Congar, Strong and Cronyn, were surprised at the assault Dr. Miner made on some of these time-honored therapeutic measures. Dr. Miner remarked that he did not desire to defend the paper, but preferred to leave the objections to it unanswered.

March 5th. Dr. Rochester reported the fact that he had never seen influenza prevail with so much of an epidemic character as at present, stating that the disease was not confined to children, but that many old people suffered from it and that in many cases it became complicated with pneumonia.

Annual meeting, April, 1867. President, Sandford Eastman; vice-president, Joshua R. Lothrop; secretary, T. M. Johnson; librarian, James B. Samo. May 7th, Dr. Gould, as retiring president, delivered an address in which he referred to the fact that during the ten years preceding, the association had lost nearly one-fourth of its members by death. Their names were as follows: Drs. Bissell, Bryant Burwell, Lewis, Newman, Sprague, Josiah Trowbridge, Treat, Wilcox, Lockwood, Howell and Butler. September 3d, Dr. Gay read a letter from Dr. Hamilton, in which he deprecated the prolonged use of splints in cases of fractures near joints, stating that it was his own custom to begin passive motion as soon as the acute inflammation had subsided, generally as early as the fourteenth day, and to lay aside the splints as soon as they could safely be dispensed with.

December 10th. Dr. Rochester moved that the secretary notify the insurance agents of Buffalo that after January 1, 1868, the fee for a certificate in life insurance as family physician in all cases would be $5, chargeable to the company for whom the examination is made.

January 7, 1868. A resolution was introduced pledging the hearty concurrence of the association in the efforts making to secure the passage of a law in the legislature, that may tend to regulate the study and practice of dentistry and to protect the people of this state from unskilful dental operations.

Annual meeting, April 7, 1868. Election of officers: President, Joshua R. Lothrop; vice-president, T. T. Lockwood; secretary, T. M. Johnson; treasurer, C. F. A. Nichol; librarian, James B. Samo. November 3d, Dr. C. Diehl was elected treasurer to fill the vacancy caused by the resignation of Dr. C. F. A. Nichol.

January 5, 1869. A committee, consisting of J. S. Trowbridge, James P. White, J. R. Lothrop, T. M. Johnson and John Cronyn, was appointed to obtain portraits of the ex-presidents of the association and of deceased members of the profession of Buffalo.

Annual meeting, April 6, 1869. Election of officers: President, Julius F. Miner; vice-president, S. W. Wetmore; secretary, T. M. Johnson; treasurer, C. Diehl; librarian, James B. Samo. May 4th, resolutions introduced by Dr. Miner were passed unanimously, directing that hereafter the fee for examination in life insurance should be $5 when made at the offices of physicians, and $2 additional when the examination is made elsewhere. Also that it should be regarded as a breach of confidence for druggists to renew prescriptions upon which is written "not to be renewed" or any words to that effect. July 6th, Dr. F. W. Abbott read a note that he intended to send to the medical profession announcing himself as a specialist in the practice of ophthalmology. Dr. White remarked that he saw no harm in his having the note forwarded to the members of the county medical society, in which opinion Dr. Miner coincided. July 22d. The death of Dr. Joshua R. Lothrop having occurred on that day it was announced and appropriate memorial resolutions were adopted.

Annual meeting, April 5, 1870. President, S. W. Wetmore; vice-president, T. M. Johnson; secretary, William C. Phelps; treasurer, C. Diehl; librarian, J. B. Samo. On motion of Dr. Rochester the secretary was authorised to procure an album to contain the photographs of the members. The portrait committee was continued, granted further time and empowered to purchase portraits of deceased members when necessary. Dr. White, in discussing a case presented by Dr. Rochester, said that he wished to bear testimony to the accuracy and acuteness of Dr. Rochester in the diagnosis of diseases of the appendix, of which he had seen many proofs before the present instance.

October 4th. Dr. Milton G. Potter reported five ovariotomies in the practice of Dr. White. Four of these cases recovered and one, which proved to be complicated with malignant disease, died.

March 7, 1871. Dr. White announced the death of Dr. J. Herman Bird, of Sioux City, Ia., formerly a resident of Buffalo. Dr. Rochester moved that Dr. Sandford Eastman, at present residing in California, and Dr. H. P. Babcock, also residing in California, be appointed delegates to the American Medical Association at its annual meeting, to be held at San Francisco, September, 1871. The motion prevailed.

Annual meeting, April, 1871. Election of officers: President, T. M. Johnson; vice-president, John Cronyn; secretary, William C. Phelps; librarian, J. B Samo.

Annual meeting, April 2, 1872. Election of officers: President, John Cronyn; vice-president, John Hauenstein; secretary, Leon F. Harvey; treasurer, B. H. Daggett; librarian, J. B. Samo. In retiring from the presidential chair Dr. T. M. Johnson took occasion to review the sanitary condition of Buffalo. He criticised the defective drainage, the condition of areas, courts, alleys, and streets, and animadverted upon the presence of pig sties, stables, manure heaps and cellars containing water and decaying vegetable matter, the Hamburg canal, which we have always with us, the milk supply and many other subjects of importance, affirming that there had been no annual report of the health physician for eight or ten years. The address attracted the attention of the association, and a committee was appointed, consisting of Drs. Strong, Rochester and Miner, to confer with the health authorities concerning the issuance of an annual report. May 7th, this committee reported that they had succeeded in accomplishing the object for which they were appointed, and asked to be discharged, which was so ordered.

Annual meeting, April 1, 1873. Election of officers: President, John Hauenstein; vice-president, J. N. Brown; secretary, L. F. Harvey; treasurer, J. J. Walsh; librarian, J. B. Samo. Dr. Cronyn remarked in retiring from the chair that he thought a live society ought to have a permanent, comfortable home, a library and hold frequent meetings. It seemed to be the opinion of many of the members present, as expressed in their remarks, that the condition of the association was one of apathy and that it was fast approaching desuetude. Dr. Samo remarked that the Society of Natural Sciences appeared to have nearly monopolised the rooms in which the meetings were held, and it would seem as though the association ought to have a room of its own.

Annual meeting, April, 1874. Election of officers: President, James P. White; vice-president, William Gould; secretary, E. R. Barnes; treasurer, J. J. Walsh; librarian, P. H. Strong. May 5th, Dr. White, on assuming the chair, stated that having been absent from the city at the time of the last meeting, when he was elected president, he had missed that opportunity to thank the association for the honor conferred. He had assisted in promoting the interests of the association for more than twenty-five years, and had taken an active part in the proceedings, and in so doing had spent many of the pleasantest hours of his life. After referring to his association with the two Flints, Hamilton, Hunt, Wilcox, Newman and others, he advocated employing a stenographic reporter, who should, under the direction of the secretary make accurate reports of the proceedings. Nothing, he said, contributed more to the success of a society than full and accurate records concerning its work. After some further general remarks concerning the importance of specialties in medicine, the regular order was taken. On motion of Dr. Miner it was ordered that hereafter the minutes be handed by the secretary to the editor of the BUFFALO MEDICAL JOURNAL for publication. November 3d, it was voted that invitations be extended to the members of the medical class at the college who are preparing to graduate at the close of the present lecture course, to be present if they desired at the regular meetings of the association.

February 9, 1875, a special meeting was held, at which there was a large attendance, to consider the prevalent excitement concerning scarlet fever. The President, Dr. White, in announcing the object of the meeting, referred to various methods by which the infection may be carried even to remote places. He cited an instance in which a woman, who had been acting as a nurse in a scarlet fever case at Mendon, where she wore a woolen dress, that she brought safely packed in her trunk, came into the family of Dr. Hunt where she also wore the dress daily. Dr. Hunt's daughter was taken sick with scarlet fever and died, not another case having occurred in the vicinity nor in the city at the time so far as known. A lengthy discussion was held, in which many members participated. A summary of several points of importance was prepared for publication in the newspapers.

Annual meeting, April, 1875. Election of officers: President, William Gould; vice-president, C. C. Wyckoff; secretary, E. N. Brush; treasurer, Joseph Fowler.

February 1, 1876.—Dr. Henry R. Hopkins read a paper relating to the sanitary authorities of Buffalo, which concluded with a resolution creating a committee of three, to be known as the sanitary committee, whose duty should be to investigate the regulations of the Buffalo health department and all matters pertaining to the sanitary government of the city, to report at the next annual meeting, with such recommendations as might be deemed necessary. The chair appointed Drs. Hopkins, Folwell and Barnes as members of the committee.

Annual meeting, April 4, 1876. Election of officers: President, C. C. Wyckoff; vice-president, E. R. Barnes; secretary, E. N. Brush; treasurer, Joseph Fowler; librarian, J. B. Samo.

Annual meeting, April, 1877. Election of officers: President, E. R. Barnes; vice-president, Thomas Lothrop; secretary, E. N. Brush. July 3d, in the course of the discussion of a paper by Dr. H. R. Hopkins on spontaneous generation, reference was made by Dr. Rochester to Prof. Lister's address on antiseptic surgery at the International Medical Congress, at Philadelphia. Dr. Miner then remarked that surgeons were constantly reporting new methods of dressing and treating wounds claiming wonderful results, yet under each of several plans—namely, the open method, the method of Lister, the hermetically sealed plan, and immersion in hot water, all so different, there were yet substantially the same results obtained. This appears to have been the first discussion of so-called Listerism—antiseptic surgery—by the association.

Annual meeting, April, 1878. Election of officers: President, Thomas Lothrop; vice-president, John Hauenstein; secretary, Joseph Fowler; treasurer, F. E. L. Brecht; librarian, P. H. Strong.

Annual meeting, April, 1879. Election of officers: President, Lucien Howe; vice-president, W. W. Miner; secretary, Joseph Fowler; treasurer, F. E. L. Brecht; librarian, James B. Samo. November 5th, Dr. Rochester read a paper on Pulmonary diseases of elevator employees. This was the first time attention had been thus publicly called to the fact that workers in elevators were especially liable to diseases of the air passages. On motion of Dr. White a committee of five was appointed, consisting of Drs. Rochester,

White, O'Brien, Davidson and Hauenstein, that should take the subject of Dr. Rochester's paper into consideration, enlist public sympathy on behalf of a reform, and if necessary strive to secure legislation on the subject. At this meeting a communication was presented from Mr. J. N. Larned, superintendent of the Buffalo Library, to the effect that the library committee desired to make purchases of books and periodicals serviceable to students and practitioners of medicine, and the opinion of the association was asked concerning the plan as well as instructions requested regarding the selection of books. On motion of Dr. Wyckoff, a committee consisting of Drs. Wyckoff, Rochester, Lothrop, Abbott and Bartlett was appointed to confer with the library committee before mentioned in regard to the choice of medical books.

December 2d, Hon. George W. Clinton read a paper on the subject of malpractice. A large number was in attendance and an interesting discussion followed.

March 2, 1880. A preamble and resolutions, relating to the Hamburg Canal, introduced by Dr. White, was adopted. The purport of the resolutions was to the effect that the common council be urged to take immediate steps toward abating the nuisance. Several persons were present who were not members of the association, among whom were Messrs. E. S. Hawley, P. P. Pratt, and Mr. Young, the city engineer. All these gentlemen concurred with the members in their views concerning the importance of obliterating the Hamburg Canal.

Annual meeting, April 6, 1880. Election of officers: President, Lucien Howe; vice-president, A. H. Briggs; secretary, Dougald Macniel; treasurer, F. E. L. Brecht; librarian, J. B. Samo. Hon. Charles Beckwith read a paper entitled, Testimony of medical experts. September 2d, Dr. William D. Granger read a paper entitled, State regulation of the practice of medicine. On motion of Dr. White a committee was appointed consisting of Drs. Granger, Barker and Keene, to obtain from some competent member of the bar a clear interpretation of the existing registration act. At a meeting held September 7th this committee reported with an opinion from Judge Clinton, whereupon a communication was sent to the Medical Society of the County of Erie, informing it of the opinion of Judge Clinton on the subject of the new medical registration law.

Annual meeting, April 5, 1881. Election of officers: President, A. M. Barker; vice-president, A. R. Davidson; secretary,

Dougald Macniel; treasurer, F. E. L. Brecht; librarian, James B. Samo.

Annual meeting, April, 1882. Election of officers: President, A. R. Davidson; vice-president, John Cronyn; secretary, C. M. Daniels; treasurer, F. E. L. Brecht; librarian, James B. Samo.

July 1, 1882. Dr. A. R. Davidson read a paper entitled, Sewer gas and its dangers. This led to considerable discussion, the members concurring with Dr. Davidson in his views concerning the bearing of sewer gas on public health.

March 6th, 1883. Dr. Julius Pohlman read a paper entitled, Geology in sanitary science, in the course of which he pointed out the relation of soil to drainage and the importance of properly constructed impervious sewers.

Annual meeting, April 3, 1883. Election of officers: President, John Cronyn; vice-president, F. W. Bartlett; secretary, C. M. Daniels; treasurer, F. E. L. Brecht; librarian, J. B. Samo.

Annual meeting, April 1, 1884. Election of officers: President, F. W. Bartlett; vice-president, Charles G. Stockton; secretary, Frederick Peterson; treasurer, F. E. L. Brecht; librarian, James B. Samo. July 15th, a resolution was passed to the effect that the Buffalo Medical Journal be requested to publish the proceedings of the association regularly and that the secretary be required to transmit to the Journal his record of each meeting. August 5th. A resolution censuring the newspaper press was passed concerning an article that had been printed in one of the morning papers, in which public attention was directed toward the private affairs of two families in the city, and which was criticised as a wanton invasion of the rights of the individual and home.

(*Continued next month.*)

